# The significance of an infant's cry: a narrative review of physiological, pathological, and analytical perspectives

**DOI:** 10.3389/fped.2025.1558951

**Published:** 2025-05-09

**Authors:** Zimai Wang, Yurui Cai, Xiaojun Wang, Shiyi Wu, Yixin Cao, Fan Xu, Min Huang

**Affiliations:** ^1^ Department of Physiology, School of Basic Medical Sciences, Chengdu Medical College, Chengdu, China; ^2^ School of Clinical Medicine, Chengdu Medical College, Chengdu, China; ^3^ Department of Evidence-based Medicine and Social Medicine, School of Public Health, Chengdu Medical College, Chengdu, China

**Keywords:** infant cry, feature extraction, infant care, somatic system disorders, neurodevelopmental and neuropsychiatric disorders

## Abstract

Infants communicate with the outside world through their cries, which often differ for various reasons. Moreover, the cries of healthy and specific pathological conditions (e.g., neurological damage) can be different. Changes in the physical and mental states can cause crying. Infant cries are characterised by a variety of features, including changes in pitch, tempo, and volume. Crying can serve as a biological indicator of an infant's health and emotions. To facilitate timely treatment, parents and caregivers can effectively understand the state of their infant by observing and identifying the characteristics of their cries. Analysis of the cries of infant with neurological disorders or severe diseases may facilitate early diagnosis of diseases and protect an infant from motor and intellectual impairments. In this article, we discuss the physiological process, causes, analysis, and application of infant cry. The purpose of this article was to fill the gap in the existing literature on the systematic integration of multi-dimensional (physiological, pathological, and psychological) analysis and deep learning applications of infant crying, and to highlight the potential of infant crying as biological indicator and in precision care.

## Background

Crying is a behavioural state in which infants express basic needs and sensations such as hunger and pain (1). Globally, about 130 million babies are born each year. Taking good care of a newborn is a huge challenge, especially for first-time parents (2). Simply following the advice of other parents and guidebooks is not enough to solve practical problems. A major issue is the difficulty new parents have in understanding the significance of infant cries. Experienced parents, caregivers, doctors, and nurses understand cries based on their experience (3). Accurately interpreting infant cries can help parents take better care of their babies. More importantly, the production of a cry requires coordination of multiple systems, and changes in one system may alter the characteristics of crying. As a result, infant cries reflect the degree of coordination of multiple organs and can be used to assess the physical condition of an infant. Therefore, it is necessary to understand the meaning behind an infant's cry.

However, infant crying is the intertwined result of multiple complex factors, which encompass the infant's age, personality traits, environmental conditions, and prior experiences. These factors interact with each other, collectively shaping the unique and variable characteristics of infant crying. Given the complexity and diversity of crying, conducting in-depth and detailed analysis faces numerous challenges, and existing analytical methods often fail to fully and accurately reveal all the information underlying it.

In recent years, published papers on infant cry have mostly focused on the in-depth analysis of its acoustic characteristics. However, there is still a lack of a comprehensive summary regarding the timing of infant crying, the physiological mechanisms underlying cry production, and the practical applications of infant crying. This narrative review synthesized evidence on infant cry research from 1968 to 2024. A comprehensive search was conducted across four major biomedical databases: PubMed, Embase, Cochrane Library, and Web of Science. The search terms are shown below: (infant cry OR baby cry OR newborn vocalization OR neonatal vocal behavior) AND (physiological analysis OR pathological indicators OR psychological correlates OR acoustic features OR deep learning OR convolutional neural networks OR biological marker OR infant care). Inclusion criteria encompassed studies investigating cry characteristics, developmental patterns, clinical correlations, or caregiver responses to infant cries. Exclusion criteria removed animal studies.

This article provides a thorough and detailed elaboration on the timing of infant crying, the physiological processes involved in cry generation, and introduces the current analytical techniques for infant crying as well as their applications in various fields. In terms of applications, this article effectively summarizes the findings in three areas: infant care, somatic system disorders and neurodevelopmental and neuropsychiatric disorders assessment, providing a more comprehensive background for understanding infant crying. This not only helps us to gain a deeper understanding of the intrinsic mechanisms of crying, but also may provide new perspectives and ideas for research in this field, promoting its development to deeper levels and broader areas.

## The general physiological process of infant cry

Complex interactions between many anatomic structures and physiologic mechanisms, that are responsible for the outcome of the infant cry (4, 5). The nasopharynx, oropharynx, laryngopharynx, and lungs make up the basic human vocal system, The lungs provide airflow for vocalisation through expansion and compression. The most important part of the vocal system is the laryngopharynx, which consists of the pharynx and vocal cords. The vocal cords have two ligamentous folds, and there is a small space between them called the vocal folds (6). The oropharynx and nasopharynx play the role of resonating cavities in the human vocal system, and the vocal tract is the entire respiratory passage from the vocal folds to the lips. The vibration of the vocal cords emits a “fundamental sound” that is extremely weak, and the sound waves must be resonated by the resonating body in order to expand and beautify the sound (7). When speaking or making a sound, the airflow exchanged at the vocal folds causes the vocal cords to vibrate, and this vibration eventually resonates through the vocal tract to produce a sound (8).

**Figure 1:**
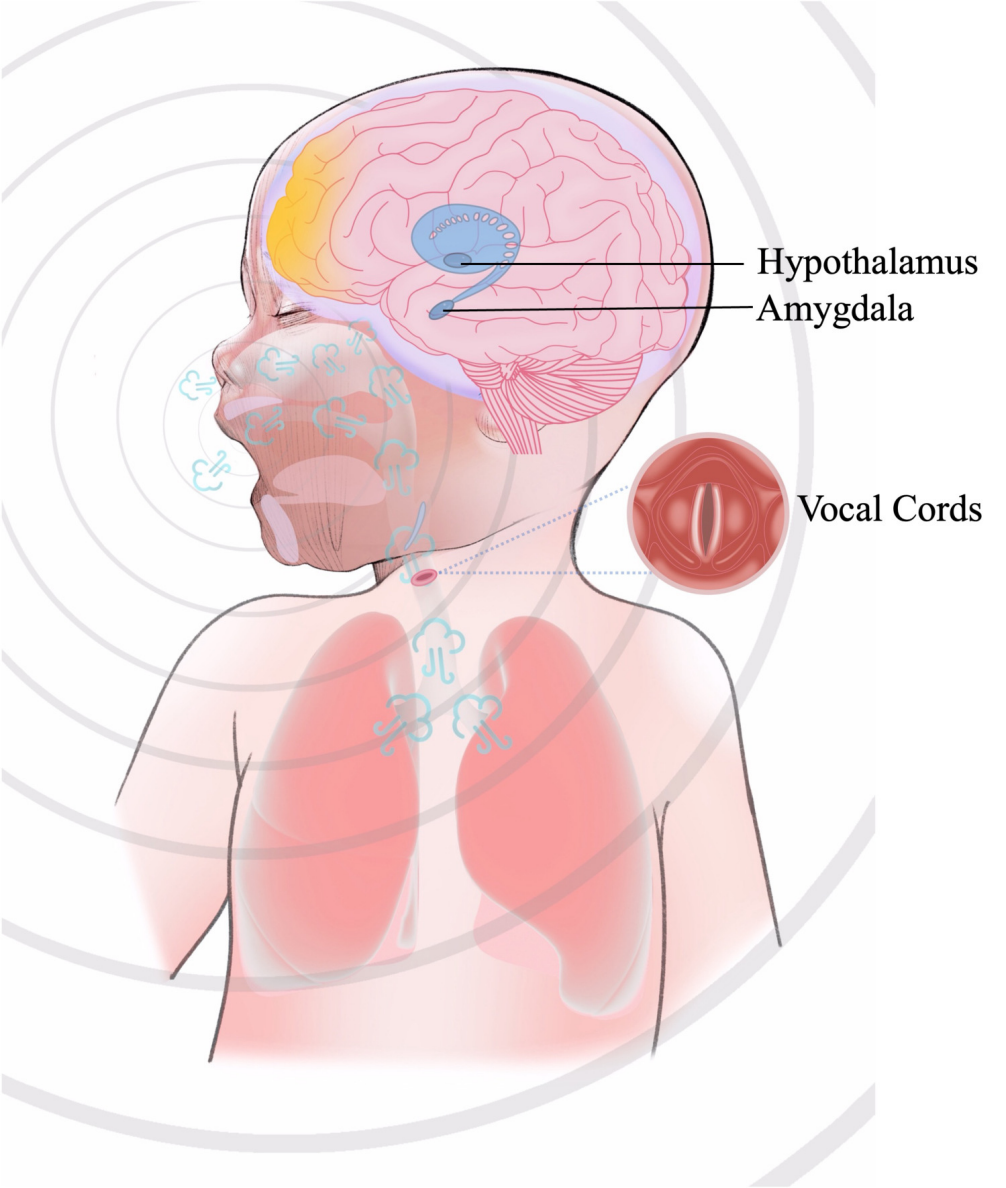
The process of crying. The production of crying originates from nociceptive or emotional stimuli that activate the amygdala within the limbic system, eliciting a negative emotional response and initiating autonomic stress responses (e.g., accelerated heart rate and respiration) via hypothalamic engagement. The hypothalamus enhances the drive of brainstem respiratory centers (medulla oblongata and pons) through neuroendocrine signaling, inducing the respiratory system (diaphragm and intercostal muscles) to generate high-velocity pulmonary airflow. This airflow traverses the larynx, inducing oscillatory vibrations of the vocal cords, whose vibratory frequency is modulated by motor neurons in the medullary nucleus ambiguus. Concurrently, acoustic resonance within the oropharyngeal and nasopharyngeal cavities amplifies and shapes the sound waves. The neurophysiological integration of these processes culminates in a vocal output characterized by affective features, reflecting the hierarchical coordination of limbic, autonomic, and brainstem motor networks.

The process of generating a cry is under the coordinated control of several brain regions (mainly the brainstem and limbic system) and requires the respiratory system. The respiratory system produces the airflow to allow vibration of the vocal folds and to make a sound. At the same time, there is resonance in all the other resonance cavities in the human body (9, 10). Crying is a type of vocalisation and a whole-body movement. As illustrated in Figure 1, the process of crying involves the central nervous system, the respiratory system, the peripheral nervous system, and various muscles. Changes in any component of this system may alter the characteristics of a cry.

The prenatal period has a significant impact on the development of the vocalization-related systems in newborns, primarily reflected in maternal health (11), nutritional status, environmental exposures (12), and psychological state (13). These aspects collectively influence the structural and functional development of systems critical for infant vocalization. Infant's vocal folds are shorter and thinner, producing sounds at higher frequencies. Adult's vocal folds are longer and thicker, vibrating at lower frequencies, which is why the frequency range of an adult's voice is typically lower (85 Hz to 255 Hz), whereas the frequency range of an infant's cry is higher (250 Hz to 700 Hz) (14). Due to these structural differences, infants produce different sounds compared with adults (15).

This difference is not only reflected in the average frequency but also in the range and concentration of the frequency distribution. The frequency range of an adult's voice is broader, covering more low-frequency components, while the frequency of an infant's cry is more concentrated and skewed towards the higher end. This difference has potential implications for sound recognition, speech processing, and acoustic communication. Understanding the fundamental characteristics of infant cries requires an analysis of both their physiological origins and their pathological deviations.

## When does an infant cry?

In the early stages, crying is primarily an expression of physiological needs, and as emotional development progresses, crying gradually becomes a form of emotional expression (16, 17). Hence, infant cries are important in determining their physical and mental states (18). Infant crying is defined as a unique behavioural state by which an infant expresses a variety of emotions, physiological needs, and their physical state. Behind the production of an infant's cry is the emotional impact of basic sensations—for example, sadness, fear, dread, or anxiety. Mood changes either in the infant itself or changes in the outside world can cause an infant to cry (3, 19).

Infants also express their physical needs through crying. When infants feel hunger, pain, restraint (from wearing clothing that is too tight), warm or cold or pressure from a foreign object, they tend to express their discomfort by crying. All the above conditions lead to normal physiological cries, and when the infant's demands are met or their discomfort is resolved, their crying will stop immediately (20).

Most importantly, an infant's cry is also an adaptive signal of distress (21). Crying in infants may be associated with one or more known diseases including infections such as sepsis (22), fever (23), deafness (24), autism (25),vomiting (23), meningitis (23), renal failure (23), respiratory distress syndrome (RDS) (26), asphyxia (23) and jaundice (27). Early diagnosis of one of these illnesses is critical to ensure timely and effective treatment, so it is important for infant caregivers and parents to understand the needs of infants through their cries. In general, an infant's crying serves as an important signal of their physical and psychological state. By analyzing the frequency and pitch of their cries, it is possible to distinguish between different needs of infants and uncover variations in their physical and emotional responses (28).

## Feature extraction of cry

The crying signals of infants differ significantly from adult speech. The variations within waveform and spectrogram of infant cries and adult speech are quite distinct, especially in terms of energy, intensity, and frequency. In the context of acoustic signals, energy refers to the total energy of the sound wave over a specified time interval, calculated as the integral of the squared amplitude of the signal. This energy metric, distinct from perceived loudness (which depends on both sound pressure level and frequency sensitivity of the human ear), can serve as an objective indicator for assessing the physiological exertion or duration of a baby's cry, potentially reflecting the degree of distress. Intensity, a measure of sound wave characteristics, is typically closely related to the baby's physical and physiological state. Fundamental frequency (F0), specifically referring to the vibration frequency of the vocal cords, is particularly significant because it provides crucial information on neural or respiratory abnormalities (29). Due to natural pauses and breathing, infant cry signals exhibit rhythmic and periodic variations. *Chittora* et al. used F_0 contour to find out the unvoiced segments from the infant cry (30).

Formants are the frequencies corresponding to prominent amplitude peaks in the sound spectrum, and their occurrence primarily depends on the shape and length of the vocal tract. Each formant corresponds to a specific resonant frequency of the vocal tract. These formants are a direct reflection of the resonant characteristics of the vocal tract during the process of sound production, with the first three typically labeled as F1, F2, and F3. The three formants F1, F2, and F3 are the most critical, as they carry most of the information in the sound, effectively distinguish between different vowels and sound features, and provide valuable information about the shape and length of the vocal tract. They are of great significance for identifying pathological features in infant cries. *Orlandi* et al. used the mean, median, standard deviation, minimum and maximum values of F0 and F1–3 to utilize the differences between full-term and preterm infant cries (31).

**Figure 2:**
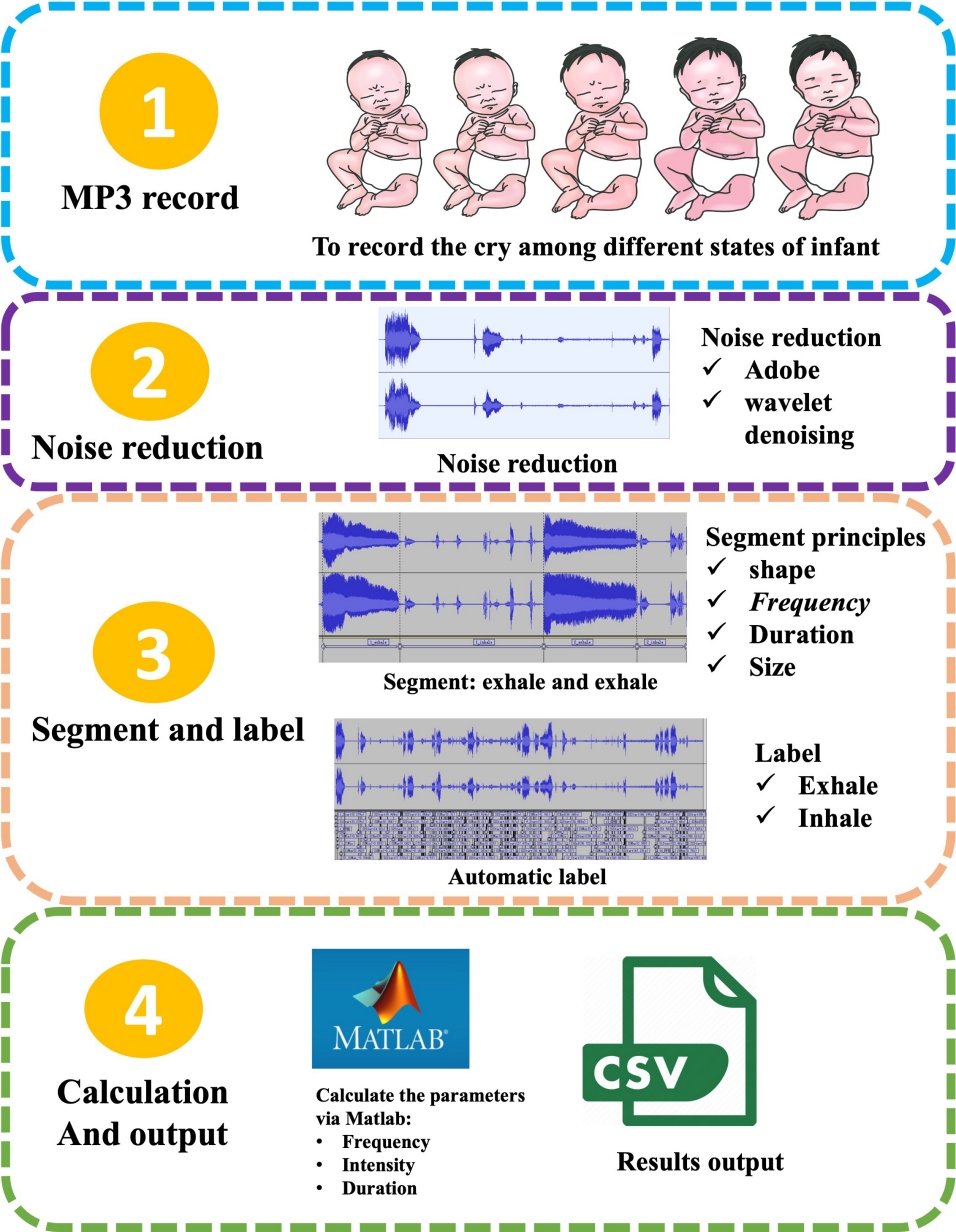
The extraction process of infant crying. (1) Collecting raw audio of infant cries under different physiological states; (2) noise reduction processing, such as employing dual-mode noise reduction technology that combines Adobe Audition® spectral editing with wavelet threshold denoising to effectively eliminate environmental noise; (3) segmentation and annotation: achieving precise segmentation of the expiratory phase based on waveform morphology, frequency characteristics, and duration thresholds, and automatically marking the inspiratory/expiratory cycles through energy envelope detection; (4) parameter calculation and output: such as using MATLAB to extract fundamental frequency, sound intensity and time-domain features, ultimately generating a structured CSV data file with timestamps.

Infant crying is a combination of various forms such as vocalization, silence, coughing, choking, and interruptions, encompassing a diversity of acoustic and prosodic information at different levels. Infant crying research involves data collection, cry signal processing, feature extraction and selection, and classification (2). Due to the sensitive nature of crying data, it is difficult for researchers to obtain the required data. Signal processing is a necessary step to remove background noise and to segment cries to create a cry database. The quality of audio data is highly dependent on signal pre-processing. This process eliminates irrelevant or unwanted information such as noise and channel distortion (32). Figure 2 provides a detailed summary of the steps involved in the extraction of infant crying.

Feature extraction is the stage where characteristics are derived from audio signals and then input into machine learning algorithms. It is one of the most crucial parts of the machine learning process. Feature extraction in the time domain or frequency domain serves as the fundamental work for cry analysis and processing. Time-domain features, such as zero-crossing rate, amplitude, and energy-based characteristics, are simple and straightforward to calculate. However, time-domain features are not robust enough to cover the variations in infant cry signals, and they are sensitive to background noise (33). Conversely, frequency-domain features possess strong capabilities to mimic the characteristics of infant cry signals. Combining prosodic features with time-domain or frequency-domain features can capture both physical and physiological information simultaneously.

Currently, commonly used features like Mel-frequency cepstral coefficient (MFCC) (33), Linear Prediction Cepstral Coefficients (LPCC) (34), and Linear Frequency Cepstral Coefficients (LFCC) (35) have proven to outperform time-domain features (33). MFCC is widely used in speech recognition as a cepstral representation of an audio signal (36). It is used by researchers to test proposed methods and is often used for baseline experiments. *Liu* et al. (37) used MFCC and two other cepstral features, LPCC and Bark Frequency Cepstral Coefficients (BFCC) to categorize the causes of infant crying. The results show that BFCC combined with neural network model can obtain the best recognition rate of 76.47%. The main idea of LPCC is to remove redundancy from the signal and try to predict the next value by linearly combining the previously known coefficients. The LFCC extraction process is like the MFCC extraction process. The difference is that it uses a linear filter -bank instead of the Mel filter-bank (38). MFCC and LPCC derive from speech recognition, and they aim at describing the phonetic structure of the signal (Table 1). However, there are significant differences between infant cries and adult speech or sounds with typical phonetic structures. For this reason, syllabic scale features have indeed proven to be more effective for infant cry detection (39).

**Table 1:** Comparison of feature extraction methods (MFCC vs. LPCC).

Dimension	MFCC	LPCC
Core Principles	Based on the auditory characteristics of the human ear, Mel-scale filtering + cepstral analysis	Based on the vocal tract model, linear predictive analysis + cepstral transformation
Key steps	Pre-emphasis → Framing → FFT → Mel Filtering → Log Energy → DCT	Pre-emphasis → Framing → LPC Coefficient Calculation → Cepstral Transformation
Mathematical formulas	$c_{n}=\overset{K}{\underset{k=1}{\sum }}\;(\log\;E_{k})\cos\;\left(\frac{n(k-0.5)\pi }{K}\right)$	$a_{i}=\arg\;\min\;\sum_{n}{\left(x(n)-\overset{p}{\underset{i=1}{\sum }}\;a_{i}x(n-i)\right)}^{2}$
	*c_n_* represents the n-th MFCC coefficient, *E_k_* represents the *k*-th output of the Mel filter bank, and *K* represents the number of Mel filters in the filter bank.	*a_i_* represents the *i*-th LPCC coefficient, *x*(*n*) represents the *n*-th sample of the speech signal, and *p* represents the order of the LPCC coefficients.
Advantages	Strong noise resistance, in line with auditory perception	Low computational load, suitable for real-time systems
Limitations	Based on the assumption of the Mel scale, high-frequency resolution is low	Sensitive to noise, assuming source-filter separation
Typical application scenarios	speech recognition, voiceprint verification	Low-resource devices, linear channel modeling

MFCC, mel-frequency cepstral coefficients; LPCC, linear prediction cepstrum coefficients; LPC, linear predictive coding.

## Current methods used to classify infant cry

Methods for classify infant cries have evolved significantly from the initial subjective perception to the objective evaluation of cries using machine learning methods, which have undergone several major breakthroughs. Research on infant cries began as early as the 1960s, based on trained nurses, *Wasz-Hockert* et al. (40) identified four types of cries: pain, hunger, birth and joy. Early research has confirmed that trained adults could auditorily distinguish between different types of cries. However, training humans to perceive infant cries is more challenging than training machine learning models, as human perception is subjective and prone to bias. In contrast, machine learning models can consistently analyze large datasets and identify subtle patterns that may be missed by human listeners (2, 41).

Researchers are beginning to explore the use of machine learning models to analyse and process sound signals. The early days relied heavily on manual feature extraction and simple machine learning algorithms for basic feature extraction and classification of cries. These algorithms learned certain feature patterns in cries from training data and tried to correlate these patterns with specific emotions or need states. These emotional states are usually determined by observing the infant's behavioral and physiological responses, and the need states are usually determined by the experience and observation of parents or caregivers (3). The establishment of basic facts about the different states usually relies on detailed observation and documentation of the infant's behavior (22). For example, when an infant cries, caregivers record the infant's behavior, physiological responses (e.g., facial expressions, body movements), and environmental factors (e.g., feeding time, diaper status) (42). To more accurately determine the underlying facts, a combination of multimodal data, such as video recordings, physiological signals (e.g., heart rate, respiratory rate), and environmental sensor data (33) These data can provide more comprehensive information to help validate the infant's emotional and need states (43). The validation process relies on the assessment of pediatricians, psychologists, and nursing specialists to determine and validate the infant's emotional and need states based on their expertise and experience and through consistency checks by multiple experts (44).

After the initial establishment of the ground truth, machine learning algorithms can be used to initially categorize the data and then compare the results with the expert assessments to further validate the accuracy of the ground truth (45). *Mukhopadhyay* et al. (46) reported that a group of people trained to recognise cries had a maximum classification accuracy of 33.09%, while machine learning algorithms based on spectral and rhythmic features could classify the same set of data with 80.56% accuracy. Thus, different types of cries can be recognised faster and more accurately by machine learning models. It should be noted that multiple studies have already emphasized the influence of the native language on the acoustic features of infant cries. This finding underscores the potential impact of language-specific factors on the ability of machine learning models to recognize infant cries (47, 48).

However, due to the complexity and diversity of cry signals, early machine learning algorithms do not have high accuracy and reliability in cry recognition and insufficient ability to process complex features. As the second winter of artificial intelligence (AI) came to an end in the 1990s (49), early deep learning models (primarily multi-layer neural networks) began gaining traction in infant cry analysis. These deep learning architectures consist of hierarchical layers of artificial neurons that simulate biological neural connectivity. A typical framework comprises: (1) input layers for signal reception, (2) hidden layers with weighted connections and activation functions, and (3) output layers generating classification predictions—forming an end-to-end computational pipeline for cry pattern decoding. Since the 2000s, methods used in infant cry research have been mainly related to scale-conjugate gradient neural networks, multilayer perceptron, general regression neural networks, evolutionary neural networks, probabilistic neural networks, neuro-fuzzy networks and time-delay neural networks (44, 50–53). In the last decade, many traditional machine learning methods, such as support vector machine (SVM), k-nearest neighbour (KNN), Gaussian mixture model (GMM), fuzzy classifier, logistic regression, k-means clustering and random forest, have been applied to classify pathological cries and the causes of cries and to detect cries (Table 2).

**Table 2:** Comparison of classification algorithms (CNN vs. SVM vs. KNN).

Dimension	CNN	SVM	KNN
Model Type	Deep Learning (Hierarchical Feature Learning)	Traditional machine learning (maximum margin classification)	Lazy Learning (Instance-Based)
Core Operations	Convolution, pooling, backpropagation	kernel techniques, convex optimization	Distance calculation, nearest neighbor voting
Feature Processing	Automatic learning of multi-level abstract features	Dependence on manual features + kernel function mapping	Features need to be manually designed
Training Complexity	High (requires GPU acceleration)	Medium [*O*(*n*^2^) ∼ *O*(*n*^3^)]	No explicit training
Inference speed	Slow (large parameter volume)	Fast (only supports vector participation in prediction)	Extremely slow (requires traversing all samples)
Interpretability	Low (black box model)	Support Vector Visualization	High (dependent on sample distance)
Data requirements	Large-scale labeled data is required	Small and medium-sized data	Small-scale data
Typical application scenarios	Image classification and time series signal analysis	Text classification, high-dimensional sparse data	Simple classification and rapid prototype validation

CNN, convolutional neural network; SVM, support vector machine; KNN, K-nearest neighbors.

In recent years, with the continuous development and improvement of deep learning technology, neural network models have been further optimized, resulting in the emergence of deep learning models including convolutional neural networks (CNN), recurrent neural networks (RNN), CNN-RNN, capsule networks, reservoir networks and neuro-fuzzy networks and others (41, 54–57). Compared to neural network models, deep learning models are more complex, with more parameters and layers. By building a deep neural network structure, the model can learn more abstract and complex feature representations, which can better process sequence data and capture temporal and frequency features in cries, improving the accuracy and robustness of cry recognition, and therefore, the ability in cry recognition and analysis has been significantly improved.

*Wang XM* et al. (58) proposed a CNN-Transformer-based model for infant crying emotion analysis, demonstrating remarkable enhancements in key performance metrics such as classification accuracy, per-class precision, and training time. *Zayed Y* et al. (59) presented a medical diagnostic system for infant crying using a combination of different audio domain features and DL algorithms. By combining spectrograms, harmonic ratios (HR) and gammatone frequency cepstral coefficients (GFCCs) and employing a deep learning process, the highest accuracy of 97.50% was achieved. *Hammoud M et al*. (33) primarily studied methods for classification and recognition of infant cries, and the results showed that using deep learning approaches and feature extraction techniques could effectively classify and recognize infant cries. Moreover, compared to traditional machine learning methods, deep learning methods demonstrated superior performance in terms of classification accuracy.

However, during the training and using of deep learning models, model errors (derived from data noise, model structure, parameter settings, training algorithms, etc.) may lead to inconsistent or conflicting results in model prediction or classification. *Zhang K* et al. proposed an improved dempster-shafer evidence theory (DST) based on wasserstein distance and deng entropy, the fusion method has a classification accuracy of 90.15%, and improves the recognition accuracy by 5.79% to 11.53% consistent with the latest methods used in baby cry recognition. The method could effectively reduce the conflict of results caused by model errors between deep learning models and improve the accuracy of infant cry recognition (60).

Besides, multimodal analysis and deep learning models can be combined with each other and work together in cry research. Multimodal analysis can extract information from multiple sources and types of data. It not only focuses on the sound features of the infant cry itself, but also considers the infant facial expressions, body movements, and possible physiological reactions when crying, which can provide more comprehensive, gain a deeper understanding of the reasons and emotional state behind infant crying (61). Multimodal analysis provides richer and more comprehensive data inputs to deep learning models, allowing the models to better understand and identify infant cries. *Laguna A* et al. (61) used multimodal analysis to collect multimodal data [i.e., crying, electroencephalography (EEG), near-infrared spectroscopy (NIRS), facial expressions, and body movements]. According to the five different conditions (i.e., hunger, sleepiness, fussiness, need to burp, and distress) defined different cry types. The study showed the robust DL algorithm named Acoustic MultiStage Interpreter (AMSI) achieved an accuracy rate of 92% in classifying infant cries. Thence, the combination of multimodal analysis and deep learning models provides more accurate tools and methods for infant cry recognition and emotion analysis.

With the improvement of computing power and the use of deep learning methods, the study of infant crying still faces many challenges. Firstly, the issue of insufficient data and scalability in research limits the further enhancement of model performance. The shared databases have limited sample sizes, and most databases are not publicly available. Current research is mostly based on datasets recorded by individual researchers, making it difficult to conduct cross-study comparisons. Additionally, ethical and legal issues in the data collection process have prevented the acquisition of data on infant crying. Secondly, there are difficulties in data collection and annotation, which is a time-consuming and labor-intensive process requiring professional expertise. To address these issues, it is necessary to combine audio acquisition and processing technologies to improve the accuracy of infant cry data collection, signal processing, and feature extraction. Through training with vast amounts of data, deep learning models can discern subtle differences in cries and more accurately determine the infant's emotions and physiological states.

## What can we learn from infant cry

The central nervous system (CNS) and vagal tone regulate the function of the laryngeal and vocal cord anatomy to produce the acoustic properties of crying, and the acoustic characteristics of infant cries may be affected by CNS pathology (10, 62). In addition, the cry signals of unhealthy infants have unique characteristics that differ from those of healthy infants because the vocal cords and respiratory system of infants are affected by certain diseases (32). Therefore, the acoustic study of infant cries is of great significance in the study of infant development. In addition to understanding the daily needs of infants, it is even more important to identify diseases by analysing pathological cry signatures, especially in wards where medical equipment and expertise are lacking (Table 3).

**Table 3:** The application of infant crying.

Supporting study	Cry feature	Application	Measures
Yamamoto et al. (63)	A 32-dimensional fast Fourier transform of sound	Recognition the needs of the baby	Accuracy: Discomfortable 30%; Hungry 92.9%; Sleepy 40%
Liang et al. (44)	MFCC	Infant emotion recognition	CNN reached up to 60% accuracy, outperforming LSTM and ANN in almost all measures.
Cabon et al. (55)	MFCC	Extraction of Premature Newborns' Spontaneous Cries	KNN: precision score 92.9%, accuracy above 90.2%; LR: recall score 94.1%, accuracy above 90.2%; MLP: precision 92.7%, recall 90.48%, accuracy 94.5%
Farsaie et al. (56)	MFCC	Development of a health diagnosis system based on infant crying	Healthy infant type (SVM-MLP with BML adaptation): FNR 8.84%, FPR 11.49% Sick infant type (PNN classifier with BML adaptation): nervous system disease: FNR 26.4%, FPR 24.6% respiratory system disease: FNR 30.5%, FPR 25.4%
Manigault et al. (43)	Short vocalizations (<500 ms) long vocalizations (≥500 ms)	The evaluation and diagnosis of neonatal opioid withdrawal syndrome	AUC 0.90, accuracy 85%, sensitivity 89%, specificity 83%
Donzelli et al. (67)	Duration, F0, F1, F2, F3, CV, PHP, MP, Cry score	To evaluate the cries of infants affected by severe protein energy malnutrition	CV f0 lower than controls (*p* < 0.0001); F1, F2, F3 lower than controls (F1: *p* < 0.0001, F2: *p* < 0.0005, F3: *p* < 0.01); MP lower than controls (*p* < 0.0001).
Orlandi et al. (31)	Mean and median of F0 median, mean, minimum maximum of F1, median and mean of F2 and F3	Classification of preterm vs. term infants	AUC 0.94, Accuracy 87.34%, Sensitivity 87.3%, Specificity 87.4%
Sheinkopf et al. (71)	Pitch (F0), Variability of pitch, Phonation, Hyperphonation, Utterance duration, Average energy/amplitude, Variability of energy/amplitude, F1, F2	Disruptions in cry acoustics may be part of an atypical vocal signature of autism in early life	At-risk infants produced pain-related cries with higher and more variable F0 than low-risk infants.
English et al. (72)	The utterance duration inter-utterance intervals loudness in dB, frication, F0.	A marker of the neurobehavioral status of newborns.	ASD infant cries were rated as more distressed, less typical, and reflecting greater pain
Mahmoudian et al. (65)	F0, F1, F2, F3, F2/F1, F3/F1, Intensity, Shimmer, Jitter, Voice break, HNR mean, Duration	The functional mechanisms of the vocal organ in hearing-impaired (HI) and normal hearing (NH) infants.	HI infants have lower intensity and higher F0 and voice break than NH infants. However, the other differences were not statistically significant.
Khozaei et al. (73)	MFCC Spectral flatness	Early screening of autism spectrum disorder	Boys: Sensitivity 85.71%, specificity 100%; Girls: Sensitivity 71.42%, specificity 100%

MFCC, mel-frequency cepstral coefficients; CNN, convolutional neural network; LSTM, long short term memory; ANN, artificial neural network; KNN, K-nearest neighbours; LR, logistic regression; MLP, multi-layer perceptron; SVM-MLP, support vector machine-multilayer perceptron; BML, boosting mixture learning; FNR, false negative rate; FPR, false positive rate; AUC, area under the curve; PNN, probabilistic neural networks; NAS, neonatal opioid withdrawal syndrome; CI, confidence interval; CV, coefficient of variation; PHP, peak harmonic proportion; MP, melodic pattern.

### Infant care aspects

Analysis of infant cries may help to identify needs such as hunger, pain and illness, leading to the development of a biological indicator or possibly a mobile app that could help parents monitor their infant's needs. *Yamamoto* et al. (63) developed a technique for recognising emotions in infants (e.g., uncomfortable, hungry or sleepy). They successfully integrated this method into a robotic baby caregiver. *Liang* et al. (44) used deep learning algorithms to recognise needs such as hunger/thirst, a diaper change, emotional needs (e.g., touching/cuddling) and pain caused by medical treatment (e.g., injection). Both CNN and long short-term memory (LSTM) models provided good performance in distinguishing between healthy and sick infants, with around 95% accuracy, precision, and recall. A CNN achieved up to 60% accuracy in determining the special needs of infants. These results could be used as metrics for future applications to help parents understand the condition and needs of their infants. *Cabon* et al. (55) established a method to extract the cries of preterm infants in noisy environments such as a neonatal intensive care unit. *Manigault* et al. (43) showed that the use of machine learning for cry analysis could improve the assessment, diagnosis and management of neonatal opioid withdrawal syndrome and contribute to standardised care for these infants.

### Somatic system disorders

Advances in the available machine learning methods have allowed researchers to automatically label normal and pathological cries, and many studies on infant cries for early diagnosis of a variety of diseases have emerged. *Saraswathy* et al. (64) reviewed 34 papers published between 2003 and 2011 on the classification of normal and pathological call signals. This included recognition of diseases such as murmuring, asphyxia, hypothyroidism, hyperbilirubinemia, and cleft palate. *Farsaie Alaie* et al. (56) demonstrated that a diagnostic system based on infant cries can distinguish between multiple neonatal diseases. They proposed a novel adaptation method known as boosted mixture learning (BML) and compared it with the traditional Bayesian adaptation method. The experimental results revealed that their proposed BML adaptation method significantly improved the system's performance. *Mahmoudian* et al. (65) found that by applying acoustic analysis of cries, the intensity, fundamental frequency and breaks of cries could be used as indicators of differentiate between infants with impaired hearing and infants with normal hearing at a very young age (less than 2 months). Early identification of hearing impairment plays an important role in the prevention of speech and language disorders.

### Neurodevelopmental and neuropsychiatric disorders

Analysis of cries from infants with neurological disorders and severe diseases, which can later lead to motor and intellectual disability, may help to facilitate early detection and timely intervention (32). Initially, *Lester* et al. (66) compared the cries of normally developing infants with those of infants who may have suffered CNS damage due to malnutrition. They showed that the cries of malnourished infants were initially longer, higher in pitch, lower in amplitude, more arrhythmic and had a longer latency to the next cry than the cries of well-nourished infants. The similarity between the cries of malnourished infants and brain-injured infants suggests that malnutrition may affect the regulatory functions of the CNS. *Donzelli* et al. (67) reached similar conclusions in a computer analysis. When *Lester and Dreher* (68) studied the effects of maternal marijuana use on the newborn cry, they found that heavy use of marijuana affected the neuropsychological integrity of infants, resulting in differences in the cry characteristics. *Lawford* et al. (69) showed that infants with underlying neuropathology have unique cries characterised by a higher fundamental frequency, dysphonia, and atypical melodies. Assessment of acoustic cry characteristics offers the potential for non-invasive and rapid point-of-care screening for neurologically high-risk infants.

The characteristics of preterm cries and their differences from those of term infants have also been explored to explain the differences observed in their neurophysiological maturation and the subsequent effects on their language development. The initial studies focused on the analysis of pain-inducing cries. The effect of neurophysiological maturity on pain-inducing cries was first revealed when *Tenold* et al. (70) found that the spectral variability of cries of term newborns was more complex than that of preterm infants. *Orlandi* et al. (31) compared the acoustic characteristics of the cries of preterm and term infants. They obtained an optimal feature set, consisting of 10 parameters, that they could use to assess the differences between preterm and term newborns with approximately 87% accuracy. Moreover, the area under the receiver operating characteristic curve reached 0.94. After comparing several machine learning models, they found that K-Nearest Neighbors (KNN) had an accuracy of up to 92.9%.

*Sheinkopf* et al. (71) studied the differences in acoustic characteristics of infant cries between high-risk infants for autism spectrum disorder (ASD) and low-risk infants. Using specialized software for analysis, they found that the fundamental frequency (F0) of pain-related cries produced by high-risk infants was higher and more variable. Especially for those high-risk infants who were later diagnosed with ASD at 36 months, their F0 values were the highest regardless of the cry type, and their cries were more poorly articulated, making it difficult to produce them in a voiced mode. The study concluded that abnormalities in the acoustic characteristics of cries may be an atypical vocal feature of autism in early infancy. *English* et al. (72) investigated parental perceptions of cries of 1-month-old infants later diagnosed with autism spectrum disorder (ASD) and non-ASD controls. Across parents, ASD infant cries were rated as more distressed, less typical, and reflecting greater pain. *Khozaei* et al. (73) developed an ASD screening method based on crying sounds and proposed a new classification approach to identify ASD features. They trained the classifier using data from children aged 18 to 53 months and tested it on ASD and TD children of different genders. The results showed that for boys, the sensitivity, specificity, and precision of the method were 85.71%, 100%, and 92.85%, respectively; for girls, these metrics were 71.42%, 100%, and 85.71%, respectively.

The relationship between infant cries and their psychological disorders can be explained through the interaction between the central nervous system and vagal tone. Vagal tone reflects the activity level of the parasympathetic nervous system, with high vagal tone typically associated with better emotional regulation, social adaptability, and physiological stability (74). Infants with higher vagal tone often exhibit more stable crying patterns, while those with lower vagal tone may demonstrate higher fundamental frequency, more irregular, or more intense cries. The central nervous system, particularly the brainstem and limbic system, regulates infants' physiological and emotional responses by modulating the activity of the vagus nerve (75). In infants with psychological disorders such as autism spectrum disorder, anxiety disorders, or depression, abnormalities may occur in the central nervous system's regulation of the vagus nerve. These abnormalities can lead to decreased vagal tone, which in turn affects the infants' emotional regulation capabilities and crying patterns.

## Challenges and perspective

In this paper, we comprehensively elaborate on the physiological process, causes, analysis, and application of infant crying. It aims to provide detailed information and valuable resources for researchers and medical professionals in this field. Despite significant advancements in infant crying research, there is still room for improvement. Given that the characteristics of infant crying are influenced by multiple factors such as the cause of crying, health status, weight, and age, the collection and analysis of crying data face numerous challenges. Therefore, integrating advanced audio acquisition and processing technologies is crucial for improving the quality of crying data collection, signal purity (especially in terms of background noise removal), and the accuracy of feature recognition. With the training of massive infant crying data, deep learning models can finely distinguish subtle differences in cries, thereby accurately determining infants' emotional fluctuations and physiological conditions.

Due to the dual pressures of strict ethical review and data scarcity, we did not analyze the cultural, ethnic, or pathological differences in demographic characteristics, nor did we validate the differences in infant crying patterns across different resource environments. Future research should proceed as follows: On the one hand, we will strictly adhere to ethical norms, ensuring that all data collection activities are approved by legitimate and compliant ethical review processes, fully respecting parents' right to informed consent, and strengthening data anonymization and privacy protection. On the other hand, we will actively explore and apply new technologies, such as federated learning and synthetic data, to effectively address the issue of data scarcity, ensuring the rational use of data resources while fully protecting personal privacy, and promoting the continuous deepening and development of research.

However, the non-specificity of crying characteristics means they cannot be directly used as a gold standard for disease diagnosis, which undoubtedly increases the complexity of differential diagnosis. Considering this, combining crying characteristics with clinical manifestations and facial expression, body movement and physiological signals (such as EEG, NIRS), can serve as an effective supplement to comprehensive pathological assessments of infants. Therefore, multimodal integration based on crying is undoubtedly an important direction for future infant crying research.

Of course, every technology is a double-edged sword. While the application of computerized tools in infant crying classification has brought many conveniences, researchers also need to be vigilant about their potential interference with normal parent-infant interaction, ensuring that the use of technology does not weaken humanized care and attention.
